# The Casket and the Ribbon

**Published:** 1849-08

**Authors:** William H. Dwinelle


*The Casket and the Ribbon, or the honors of Ether.* By WILLIAM H. DWINELLE, M. D.

A pamphlet bearing the above title, republished from the American Journal of Dental Science, has been sent to us. To understand the significancy of the title, the reader should be informed that Dr. Morton, some time ago, received, as a mark of gratitude for his discovery of the anæsthetic virtues of the inhalation of ether, a casket containing a thousand dollars. More recently, Dr. Jackson, for the same discovery, is reported to have received the insignia of the Legion of Honor of France. The work is a new addition to the bibliography of the interminable ether controversy, and its object is to advocate the merits of the Morton party. The whole matter of this controversy lies in a small compass. The Jackson party aver that Morton got the idea from Jackson. The Morton party, on the other hand, deny that Jackson originated the idea, and that Morton only applied to Jackson, as a chemist, for some information involved in making the discovery. The point at issue is one of honor and veracity, which cannot be satisfactorily settled by the testimony of others, or circumstantial evidence. Both parties once made oath that it was a joint discovery. Whether it was so, or not, in their own estimation, respectively, must be left as a matter of consciousness and conscience with them. It is useless to argue the subject. Prolonged controversy will only serve to render it more and more complicated and confused. There may, however, be a Providence in this, for we honestly believe, from what examination we have given to the matter, that the honor of the discovery belongs to Wells. With reference to the rival claims of the two Boston competitors, the following questions suggest themselves: if, as the partizans of Jackson contend, he anticipated Morton in the idea, why did he neglect to verify it by experiment, or, at least, to promulgate it for the benefit of humanity, as well as his own honor? If he entertained any adequate sense of the importance of the discovery, why keep it locked in his own breast? Had he experienced a fraction of the anxiety respecting its paternity *before* Morton's experiments, which he appears to have felt *afterward*, would the question of authorship have been as difficult to settle as it now is? We throw out these queries because they occur to us in looking over the pamphlet the title page of which we have given. We have no bias to either side. Whatever credit may belong to either or both the rival candidates, for rendering the value of anæsthetic inhalations appreciable and available, we have no doubt that the ultimate verdict of mankind will be, that to neither of them is the priority of discovery justly due.

